# A Game Model for Analyzing Wireless Sensor Networks of 5G Environment Based on Adaptive Equilibrium Optimizer Algorithm

**DOI:** 10.3390/s23198055

**Published:** 2023-09-24

**Authors:** Weimin Zheng, Fanying Meng, Ning Liu, Shuo Huang

**Affiliations:** College of Computer Science and Engineering, Shandong University of Science and Technology, Qingdao 266590, China; zhengwm901@126.com (W.Z.); sdust_mfy0120@163.com (F.M.); huangshuo1030@163.com (S.H.)

**Keywords:** 5G, wireless sensor networks, game model, adaptive equilibrium optimization, Nash equilibrium

## Abstract

Wireless sensors networks (WSNs) play an important role in life. With the development of 5G, its security issues have also raised concerns. Therefore, it is an important topic to study the offense and defense confrontation in WSNs. A complete information static game model is established to analyze the offense and defense confrontation problem of WSNs in 5G. An adaptive equilibrium optimizer algorithm (AEO) based on parameter adaptive strategy is proposed, which can jump out of the local optimal solution better. Experiments show that the optimization ability of AEO outperforms other algorithms on at least 80% of the 23 classical test functions of CEC. The convergence speed of AEO is better in the early stage of population iteration. The optimal offensive and defensive strategy under different offense and defense resources through simulation experiments is analyzed. The conclusion shows that when the offensive resources are large, the offender takes an indiscriminate attack. When the defense resources are small, the defender should defend the most important elements, and when the defense resources are large, the defender should allocate the same resources to defend each element to obtain the maximum benefit. This paper provides new solution ideas for the security problems under the offense and defense game in WSNs.

## 1. Introduction

The networks of 5th Generation Mobile Communication Technology (5G) have developed rapidly in recent years. Due to its characteristics, such as high speed, low latency, and large scale [1,2], 5G network technology is widely used in all aspects of life, such as healthcare, agriculture, and communication [3,4,5,6]. Wireless sensor networks (WSNs) are the smallest units of networks, and they can support large-scale deployments, high reliability, and high mobility [7]. WSNs are more broadly applied in the 5G network environment [8,9,10]. WSNs are increasingly important in the field of communication technology [11]. For example, Internet of Things (IoT) applications of WSNs over 5G infrastructure addressed by Martinez successfully reduce the latency of exchanging information between facilities [12]. Dhinakaran creates a hybrid clustering and routing strategy for data aggregation in a 5G WSN. This strategy makes the network lifetime longer [13].

Wireless communication has become an integral part of the mobile network. As the demand for 5G mobile wireless networks grows [14], the number of devices and service types is rising. Therefore, the security threat landscape of WSNs in 5G has also grown significantly [15,16]. Rishita discusses the security issues and challenges in WSNs and elaborates on the attack behaviors that WSNs are vulnerable to at the network layer [17]. Currently, many hackers adopt the Advanced Persistent Threat (APT) offense model [18,19]. Denial-of-service offense mode (DOS) in computer network broadband and connectivity offense [20] attack the target nodes and information transmission paths in the target network. In the face of various offense patterns of hackers, it is particularly important to maintain the security of WSNs.

Many scholars apply the offense and defense game model to network security and some information transmission resource allocation problems [21,22,23,24]. Nowadays, many scholars use game theory to solve a series of security problems in WSNs. Maryam uses a Bayesian game model to make a secure routing protocol, which can improve the detection accuracy of an intrusion detection system (IDS) in WSNs [25]. Sudha combines software-defined networks with Stackelberg games to achieve the optimal data offloading problem in 5G [26]. Zhou analyzes the micro-mechanism of malware propagation in WSNs from the perspective of game theory, which can be of guiding significance for inhibiting the spread of malware [27]. The combination of game theory methods and WSNs can better address a series of security problems existing in WSNs. However, with the development of large-scale and high-performance 5G, the development and extension of WSNs have diversified. The existing attack methods and scale of offense and defense are no longer sufficient for the environment in which they are located. The offense and defense strategies in WSNs under the 5G environment are no longer in a singular form but rather align with the characteristics of large-scale deployments in 5G. Regarding offense and defense of WSNs in 5G, most of the attack methods are to attack the target node while ignoring the attack on the information transmission link. The problem of how to analyze the large-scale offense and defense confrontation behaviors of WSNs in 5G has become an important issue to be studied. Therefore, the main contributions of this paper are as follows.

(1) A network topology diagram based on WSNs is constructed. The nodes and the transmission links in the WSN are abstracted as nodes and edges in an undirected graph. The scale of the offense and defense confrontation is expanded, and the adversarial game is carried out on the graph structure.
(2) A complete information static game model is established for the offense and defense problem in WSNs. And the existence of Nash equilibrium in the model is proved. Therefore, offense and defense game behaviors of WSNs in the 5G environment can be analyzed more clearly and intuitively.
(3) An adaptive strategy is applied to an equilibrium optimizer algorithm (EO), and an adaptive equilibrium optimizer (AEO) algorithm is proposed to improve the optimization ability of EO. And AEO is applied to the problem of solving Nash equilibrium under mixed strategies.
(4) The behavior process and strategy choices of both offenders and defenders under different attack and defense resources are discussed and analyzed.

The paper is structured as follows. Section 2 discusses the related work in this paper. Section 3 establishes the offense and defense game model with respect to the established network topology graph. Section 4 proposes the AEO to solve the Nash equilibrium under mixed strategies in this game model. Section 5 conducts simulation experiments to derive the mixed strategy adopted by both offenders and defenders under different resources. Section 6 gives conclusions.

## 2. Related Work

This section introduces the application and development of game theory in network security and the ideas and steps of the EO.

### 2.1. Application of Game Theory to Network Security

Game theory describes a multi-player decision-making scenario as a game. Each player chooses the action that gives him or her the best payoff while predicting the rational actions of the other players [28]. Regarding the time-series nature of behavior, game theory is subdivided into two categories, namely static games and dynamic games. In terms of whether there is cooperation between participants, games can be divided into cooperative games and non-cooperative games. For the problem of cyberspace security, many scholars have used game theory to solve it and have achieved certain results [29]. Afrand studies the offense and defense game problem in WSN intrusion detection during 2004–2005. He establishes a non-cooperative game model for offenders and defenders and constructs the payoff function and Nash equilibrium in this game. The chance of detecting an intrusion can be significantly improved through the game [30,31,32]. Han applies a non-cooperative static information game model to intrusion modeling in WSNs, which improves prediction accuracy and reduces the energy consumption of IDS [33]. Shamik applies a non-cooperative imperfect information game to the distributed sensor network power control problem. He obtains the maximum payoff of the model by analyzing the Nash equilibrium [34]. Liu combines intelligent computing with Stackelberg game to analyze the attack and defense adversarial behavior under a graph structure network [35]. Yang proposes a WSN offense and defense game model for multiple crimes. The game process of WSN under three modes of external offense, internal offense, and hybrid offense, respectively, gives practical guidance for the design of an intrusion detection system in WSNs [36]. Deyu proposes a novel routing protocol based on evolutionary game theory to improve energy efficiency and longevity of WSNs [37]. Yenumula uses a zero-sum game approach to detection to build a framework and detect malicious nodes of nodes in the forward data path to improve the defense of WSNs [38]. It can be seen that many scholars have applied game theory to WSN security. Game theory is also applied for the offense and defense problem of WSNs in this paper. Intelligent calculation is used to solve and analyze the Nash equilibrium problem in the established game theory model to improve the solution accuracy.

### 2.2. Equilibrium Optimizer Algorithm

Heuristic algorithms are proposed relative to optimization algorithms. Scholars have proposed heuristic algorithms such as Bat Algorithm (BA) [39], Differential Evolution algorithm (DE) [40], Particle Swarm optimization algorithm (PSO) [41], and Whale Optimization Algorithm (WOA) [42,43]. These algorithms have improved the ability to search for optimal solutions. EO is a physics-based heuristic optimization algorithm for dynamic source and sink models proposed by Afshin in 2020, which has the advantages of good optimization and fast convergence [44]. The heuristic algorithm has also been improved by adding many strategies. Zheng presents a Levy flight black edge regeneration black algorithm (LEBH) to speed up the convergence rate of BH [45]. Zheng applies the compact strategy to the snake optimization algorithm (SO). The compact snake optimization algorithm (cSO) is proposed, which effectively reduces the use of memory resources [46]. Wang proposes the adaptive Bat algorithm (ABA), which can dynamically and adaptively adjust the flight speed and direction, significantly improving the global convergence accuracy of the BA [47]. Zhan applies the adaptive optimization strategy to the PSO (APSO). The problem of slow convergence of PSO and ease of falling into the local optimal land was effectively solved. [48]. Ahmed and Qin also apply adaptive strategy to WOA (ANWOA) and DE (ADE), respectively, and the convergence speed and optimization accuracy of the original algorithm can be effectively improved [49,50]. The adaptive strategy can dynamically adjust the parameters of the algorithm and change the direction and speed of particle motion in the algorithm so that it can easily solve the problem that the algorithm is prone to local optimization and improve the accuracy of the global optimization. Thus, the adaptive strategy is applied to EO to improve the optimization ability and convergence speed of EO.

The main of inspiration for the EO is the simple mixing of well-defined dynamic mass balance phenomena on the control volume. The first-order ordinary differential equation for the mass balance equation is given by Equation (1) [44].

(1) $$V\frac{dC}{dt}=QC_{eq}-QC+G$$

$V\frac{dC}{dt}$ is the rate of mass change in the control volume, and *C* is the concentration inside the control volume. When $V\frac{dC}{dt}$ is equal to zero, the solution reaches a steady state. *Q* is the volumetric flow rate into and out of the control volume, and $C_{eq}$ represents the concentration at an equilibrium state. *G* is the mass generation rate inside the control volume. By solving for Equation (1) [44], through the arrangement and combination of Equation (1) [44], $\frac{dC}{dt}$ can be converted into a function of $\frac{Q}{V}$. $\lambda =\frac{Q}{V}$ is introduced into the formula as the flow rate, and *C* can be expressed in the form of another Equation (2) [44]. *F* is the coefficient of the exponential term, which can be calculated by Equation (3) [44].

(2) $$C=C_{eq}+\left(C_{0}-C_{eq}\right)F+\left(1-F\right)\frac{G}{\lambda V}$$

(3) $$F=exp\left[-\lambda \left(t-t_{0}\right)\right]$$

λ is the mobility rate, and $C_{0}$ is the initial concentration of the control volume at the initial time $t_{0}$. The three parts of Equation (2) [44] can represent the three update rules in the inspired EO. The first is the equilibrium concentration, and the second is related to the concentration difference and represents the search mechanism. The third represents the part of the optimal solution. Applying Equation (2) [44] to the EO, *C* represents the solution obtained in the current iteration, and $C_{eq}$ represents the optimal solution in the current generation. Thus, the EO continuously updates the positions of the particles through iterative search and searches for the optimal solution through a combination of local search and global search. The principle and process of the EO are shown below.

The initial concentration is constructed based on the number and dimensional of the particle swarm. The particle swarm is initialized as in Equation (4) [44].

(4) $$C_{i}^{0}=C_{min}+randi\left(C_{max}-C_{min}\right),i=1,2,\ldots ,n$$

$C_{i}^{0}$ represents the initial concentration of the *i*th particle, and it also represents the initial position of the *i*th particle. $C_{max}$ and $C_{min}$ denote the minimum and maximum values of the range. *n* represents the number of particle groups, and randi is a random number in the range of [0, 1].

In each iteration, each particle randomly selects a particle in the equilibrium state pool with the same probability to update its concentration. The equilibrium state pool is defined by the following Equation (5) [44].

(5) $$C_{eq,pool}=\left\{C_{eq1},C_{eq2},C_{eq3},C_{eq4},C_{eq5})\right\}$$

$C_{eq1}$, $C_{eq2}$, $C_{eq3}$, and $C_{eq4}$ are the best four solutions obtained throughout the current iteration. $C_{eq5}$ represents the average position of the four solutions.

To optimize the search ability, two parameters $a_{1}$ and $a_{2}$ are introduced to improve Equation (3) [44] to better balance the local and global search. The improved equation is given in Equation(6) [44], where *t* is defined as a function of iteration (Iter) and it decreases as the number of iterations increases, as shown in Equation (7) [44].

(6) $$F=-a_{1}sign\left(r-0.5\right)\left(e^{-\lambda t}-1\right)$$

(7) $$t={\left(1-\frac{Iter}{MaxIter}\right)}^{a_{2}\frac{Iter}{MaxIter}}$$

*r* and λ are random variables in the range of [0, 1]. The $a_{1}$ in Equation (6) [44] represents the control exploration capability. The larger $a_{1}$ becomes, the greater the exploration capacity and the weaker the exploitation capacity. The $a_{2}$ in Equation (7) [44] represents the managed exploration capacity. The larger the $a_{2}$, the greater the exploitation capacity and the weaker the exploration capacity. $sign\left(r-0.5\right)$ affects the direction of exploration and development.

Generation rate (*G*) is one of the most important terms in EO, providing precise solutions by improving the development phase. G is described as a first-order exponential decay process, which is used in many engineering applications, as shown in Equation (8) [44].

(8) $$G=G_{0}e^{-k\left(t-t_{0}\right)}$$

$G_{0}$ is the initial value and *k* is the attenuation constant. To better adapt to the iteration of the algorithm, the exponential term of Equation (8) [44] is adopted. The generation speed control parameter G·GP is defined as Equation (9) [44]. *G* is the mass generation rate, defined as Equation (10) [44]. Combined with Equation (8) [44], G is defined in EO as shown in Equation (11) [44], which can provide an exact solution by improving the development phase.

(9) $$G\;\cdot \;GP=\left\{\begin{array}{cc} 0.5r_{1} & r_{2}\geq GP\\ 0 & r_{2}\leq GP \end{array}\right.$$

(10) $$G_{0}=G\;\cdot \;GP\left(C_{eq}-\lambda C\right)$$

(11) $$G=G_{0}F$$

GP=0.5 gives an ideal balance of local and global search capabilities.

In summary, the rules for updating the particle positions in the EO are given in Equation (12) [44].

(12) $$C=C_{eq}+\left(C-C_{eq}\right).F+\frac{G}{\lambda V}\left(1-F\right)$$

Equation (12) [44] is divided into three terms, the first term being the equilibrium concentration. The second and third terms indicate the change in concentration. The second term can use the concentration difference to search globally to find the best solution. The third part can make the solution more precise when the solution is found. This provides better global and local search based on the difference of symbols of the second and third terms.

Algorithm 1 is the pseudo-code for the EO.
**Algorithm** **1** Equilibrium Optimizer**Require:** ParticleNumber, MaxIter, $C_{max}$, $C_{min}$ **Ensure:** Best Position 1:Initialize the position of the particle swarm using Equation (4)2:Construct the fitness function Fit3:Initialization parameters $a_{1}=2$, $a_{2}=1$, GP=0.54:**for** iter = 1: MaxIter **do**5:    Find the location and concentration of the top 4 best adapted particles $Ceq_{1},Ceq_{2},Ceq_{3},Ceq_{4}$.6:     $C_{eq5}=\left(C_{eq1}+C_{eq2}+C_{eq3}+C_{eq4}\right)/4$7:     $C_{eq,pool}=\left\{C_{eq1},C_{eq2},C_{eq3},C_{eq4},C_{eq5}\right\}$8:     $t={\left(1-\frac{Iter}{MaxIter}\right)}^{a_{2}\frac{Iter}{MaxIter}}$9:     **for** i = 1: ParticleNumber **do**10:            Randomly select a candidate $C_{eq}$ from $C_{eq,pool}$11:            Generate random vectors λ and *r*12:            Use Equations (6)–(11) to calculate *F*, G·CP, $G_{0}$ and *G*.13:            Update $C_{i}=C_{eq}+\left(C-C_{eq}\right).F+\frac{G}{\lambda V}\left(1-F\right)$14:      **end for**15:      iter = iter+116:**end for**17:Best Position = $C_{eq1}$


## 3. Offense and Defense Game Model

An abstract model of the topology graph of WSNs is presented in this section. The elements under offense and its importance are defined and calculated. In addition, the complete information static game model is presented. And the development of offense and defense strategies and payoff functions to provide rules for the offense and defense game is shown in this section.

### 3.1. Network Topology Diagram Model

**Figure 1:**
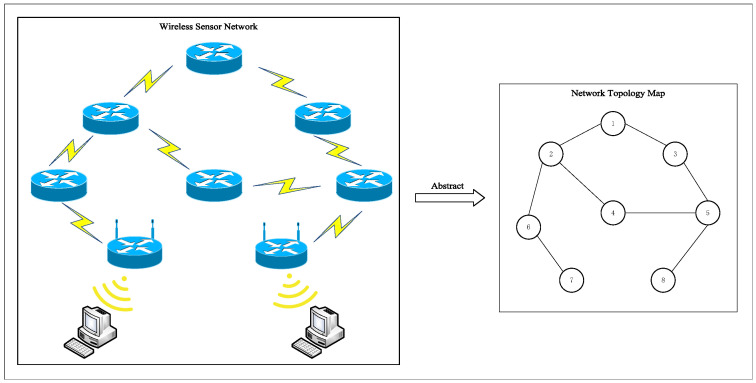
Illustration of the process of abstracting a WSN into an undirected graph.

A simple undirected graph $G\left(V,E\right)$ can be seen as an abstraction of a WSN, where $V=\left\{V_{1},V_{2},\ldots ,V_{N_{v}}\right\}$ is a set of nodes. $N_{v}=\left|V\right|$ is the total number of nodes. Each $V_{i}$ represents a sensor node in a WSN. $E\subseteq V\times V=\left\{E_{1},E_{2},\ldots ,E_{N_{e}}\right\}$ is a set of edges, where $N_{e}$ is the total number of edges. And each $E_{i}$ represents a transmission link through which data can be transmitted between two sensor nodes. The mapping abstraction is shown in the following Figure 1.

$A{\left(a_{ij}\right)}_{N_{e}\times N_{e}}$ is the diagonal matrix of graph *G*, and $a_{ij}$ represents the presence or absence of link connectivity between node $V_{i}$ and $V_{j}$. If nodes $V_{i}$ and $V_{j}$ have a message transmission link and assume that the number of packets transmitted, received, and forwarded in the link is $S_{ij}$, and $S_{ij}=S_{ji}$, then the link is assigned a weight $a_{ij}=a_{ji}=S_{ij}=S_{ji}$. Otherwise, $a_{ij}=a_{ji}=0$. Therefore, the definition of diagonal matrix *A* is defined in this paper as follows in Equation (13).

(13) $$a_{ij}=a_{ji}=\left\{\begin{array}{cc} S_{ij}=S_{ji} & \mathrm{Node}\;i\;\mathrm{is}\;\mathrm{connected}\;\mathrm{to}\;\mathrm{node}\;j\\ 0 & \mathrm{Node}\;i\;\mathrm{is}\;\mathrm{unconnected}\;\mathrm{to}\;\mathrm{node}\;j \end{array}\right.$$

Since the total number of packets transmitted, received, and forwarded by each node is not equal, the importance of each node in a WSN is different. $I_{V_{i}}$ represents the importance of node $V_{i}$. The definition is shown in Equation (14).

(14) $$I_{V_{i}}=a_{i1}+a_{i2}+\cdots +a_{iN_{v}}$$

Similarly, each edge has a different level of importance. $G_{max}$ is the maximum connectivity of the undirected graph *G*. $G_{E_{i}}$ is the maximum connectivity of the graph after removing an edge $E_{i}$. Assume that $E_{i}$ connects node $V_{i}$ with node $V_{j}$. Then $I_{E_{i}}$ is the importance of that $E_{i}$ edge, defined as in Equation (15). The calculation of link importance is divided into two parts. The first part is the proportion of the number of packets transmitted, forwarded, and received by the edge to the total number of packets transmitted, forwarded, and received by the whole network. The second part is the size of the change of the graph connectivity after removing the link. The larger it is, the more important the link is.

(15) $$I_{E_{i}}=\frac{a_{ij}+a_{ji}}{\sum_{i=1}^{N_{v}}\sum_{j=i}^{N_{v}}aij}+\frac{G_{max}-G_{E_{i}}}{G_{max}}$$

### 3.2. Offensive–Defensive Strategies

According to the model established in this paper, offensive and defensive strategies are formulated, as shown in the following four points.

(1) Both nodes and edges can be attacked in this model. The costs of attacking and defending each node and edge are the same.
(2) The game is a complete information static game. Both offenders and defenders have full information about the network topology graph.
(3) The game is played for one round and there is only one player in each role of the game, and both players act simultaneously.
(4) In each round of the offense and defense game, each node and edge can only be attacked once. When an offense on a node is successful, both the node and its connected edges are deleted. When an offense on an edge is successful, only that edge is deleted.

$Q=N_{e}+N_{v}$ is the total number of offensive and defensive resources. $Q_{A}$ represents the total number of resources that the offender can use for offense. $Q_{D}$ represents the total number of resources that the defender can use for defense. $Q_{A}$ and $Q_{D}$ are less than or equal to *Q*.

$S_{A}$ and $S_{D}$ represent the set of strategies for offense and defense, respectively. $\left|S_{A}\right|$ and $\left|S_{D}\right|$ represent the respective number of strategies that are calculated by permuting $C_{Q}^{Q_{A}}$ and $C_{Q}^{Q_{D}}$. The calculation is shown in Equation (16).

(16) $$\left\{\begin{array}{c} M=\left|S_{A}\right|=C_{Q}^{Q_{A}}\\ N=\left|S_{D}\right|=C_{Q}^{Q_{D}} \end{array}\right.$$

Define one of the offensive strategies as $s_{a}=\left[s_{a1},s_{a2},\ldots ,s_{aQ_{A}}\right]$, and one of the defensive strategies as $s_{d}=\left[s_{d1},s_{d2},\ldots ,s_{dQ_{D}}\right]$. Assuming that node $V_{i}$ is attacked, then $s_{ai}=1$; otherwise, $s_{ai}=0$. Similarly, if node $V_{i}$ is defended, $s_{di}=1$; otherwise, $s_{di}=0$. For this, AV is a set to represent the state of each point being attacked. As shown in Equation (17), $AV_{i}=1$ represents that the *i*-th node is successfully attacked; otherwise, $AV_{i}=0$.

(17) $$\left\{\begin{array}{cc} A_{V_{i}}=1, & s_{ai}=1\&s_{di}=0\\ A_{V_{i}}=0, & \left(s_{ai}=1\&s_{di}=1\right)\|s_{ai}=0 \end{array}\right.$$

Whether the edge is successfully attacked is also the same as the above method of the node. When the game is complete, the network topology diagram at this point is defined as $G^{{}^{\prime }}$, and the maximum connectivity of this network topology diagram at this time is $G_{max}^{{}^{\prime }}$.

### 3.3. Payoff Function

The payoff function is used to calculate the payoff of players under different strategies. $U_{A}$ and $U_{D}$ are the set of revenue of the offender and the defender under different offense and defense strategies. $U_{A}\left(s_{a},s_{d}\right)$ denotes the gain under the strategy $s_{a}$ of the offender and the strategy $s_{d}$ of the defender. The equation for calculating $U_{A}\left(s_{a},s_{d}\right)$ is Equation (18).

(18) $$U_{A}\left(s_{a},s_{d}\right)=\frac{\sum_{i=1}^{|N_{e}|}\left(AV_{i}\right)\times I_{V_{i}}}{\sum_{i=1}^{|N_{V}|}I_{V_{i}}}+\frac{G_{max}-G_{max}^{{}^{\prime }}}{G_{max}}$$

The gain of the offenders comes from two parts. The first part is the gain from attacking each node. The second part is the change in the maximum connectivity of the graph after completing all offenses. This game model is a zero-sum model of a complete information static game, so the gain of the defender can be calculated by Equation (19). The revenue matrix is shown in Table 1.

(19) $$U_{D}=-U_{A}$$

### 3.4. Offense and Defense Game Model

A model of the offense and defense game $GM=\left(A,D,S_{A},S_{D},U_{A},U_{D}\right)$, which is a complete information zero-sum static game model. The goal of the offender is to maximize his own gain by attacking in the case of $Q_{A}$ resources. It can be shown by Equation (20).

(20) $$\begin{array}{c} \max\;U_{A}\left(s_{a},s_{d}\right)\\ s.t\;s_{a}\in S_{A}\\ \;\;\;\sum_{i=1}^{|N_{v}|}s_{ai}=Q_{A}\\ \;s_{ai}=0,1 \end{array}$$

The goal of the defender is to minimize the gain of the offender by protecting $Q_{D}$ resources in the network. It can be shown by Equation (21).

(21) $$\begin{array}{c} \min\;U_{A}\left(s_{a},s_{d}\right)\\ s.t\;s_{d}\in S_{D}\\ \;\;\;\sum_{j=1}^{|N_{v}|}s_{dj}=Q_{D}\\ \;s_{dj}=0,1 \end{array}$$

The game is played between the offender and defender, and a strategic equilibrium is reached. Therefore, there is a Nash equilibrium under pure strategy and a Nash equilibrium under mixed strategy in this game model. $S_{A}^{*}$, $S_{D}^{*}$ are assumed to be optimal offense and defense strategies under the offense and defense game. The Nash equilibrium in this game model must satisfy Equation (22).

(22) $$\left\{\begin{array}{cc} U_{A}\left(S_{A}^{*},S_{D}^{*}\right)\geq U_{A}\left(S_{Ai},S_{D}^{*}\right) & \forall i\in |S_{A}|,S_{Ai}\in S_{A}\\ U_{D}\left(S_{A}^{*},S_{D}^{*}\right)\geq U_{A}\left(S_{A}^{*},S_{Dj}\right) & \forall j\in |S_{D}|,S_{Dj}\in S_{D} \end{array}\right.$$

## 4. Game Solution

This section solves the Nash equilibrium for the offense and defense game model proposed in this paper. The solution steps are proposed in terms of pure and mixed strategy Nash equilibrium. The EO is improved from three aspects, the AEO is proposed, the effectiveness of the proposed algorithm is verified, and the AEO is used to solve the Nash equilibrium.

### 4.1. Pure Strategy Nash Equilibrium

The min–max theorem is applied to the solution of Nash equilibrium under pure strategies [51]. Strategies under Nash equilibrium make it unprofitable for any participant to deviate unilaterally from their equilibrium strategy. Assuming that the strategy $\left(S_{A}^{*},S_{D}^{*}\right)$ satisfies Equation (23), it is a Nash equilibrium under a set of pure strategies in the game.

(23) $$\min_{1\leq i\leq M}\max_{1\leq j\leq N}U_{A_{ij}}=\max_{1\leq i\leq M}\min_{1\leq j\leq N}U_{A_{ij}}=U_{A_{i^{*}j^{*}}}$$

The idea of the theory is to find the optimal strategy when in a bad situation. When one player in the game offers a choice of strategies, the other player will choose the strategy that maximizes their gain. And they give feedback on the strategy to the first player. The first player also compares whether the choice is optimal for this strategy. If the combination of strategies is optimal for both players, a Nash equilibrium is reached.

### 4.2. Mixed Strategy Nash Equilibrium

A mixed strategy assigns a probability to each pure strategy. $P_{A}=\left(P_{A1},P_{A2},\ldots ,P_{AM}\right)$ is assumed to the probability to the offender taking each strategy. $P_{D}=\left(P_{D1},P_{D2},\ldots ,P_{DN}\right)$ is the probability that the defender takes each strategy. For offenders and defenders adopting mixed strategies, the sum of the probabilities of their choosing different strategies satisfies Equation (24).

(24) $$\sum_{i=1}^{M}P_{Ai}=\sum_{j=1}^{N}P_{Dj}=1$$

There are two ways to solve the Nash equilibrium for a mixed strategy, the first being that both sides wish to maximize their benefits under the mixed strategy. Equation (25) is the expected value of the benefits that both sides of the offense and defense game wish to achieve. The second way is that the optimal mixed strategies of both sides of the game will give the opponents equal expected benefits under the different strategies they choose, as shown in Equation (26).

(25) $$\begin{array}{cc} \; & U_{A}^{{}^{\prime }}=max\left(\sum_{i=1}^{M}\sum_{j=1}^{N}P_{Ai}\;\cdot \;P_{Dj}\;\cdot \;U_{A}\left(S_{Ai},S_{Dj}\right)\right)\\ \; & U_{D}^{{}^{\prime }}=max\left(\sum_{i=1}^{M}\sum_{j=1}^{N}P_{Ai}\;\cdot \;P_{Dj}\;\cdot \;U_{D}\left(S_{Ai},S_{Dj}\right)\right)\\ \; & \;\;s.t.\;\left\{\begin{array}{c} \sum_{i=1}^{|N_{v}|}s_{ai}=Q_{A}\\ \sum_{j=1}^{|N_{v}|}s_{dj}=Q_{D}\\ \sum_{i=1}^{M}P_{Ai}=1\\ \sum_{j=1}^{N}P_{Dj}=1 \end{array}\right. \end{array}$$

(26) $$\begin{array}{c} \sum_{i=1}^{M}p_{Ai}\;\cdot \;U_{D}\left(S_{A_{i}},S_{D_{1}}\right)=\sum_{i=1}^{M}p_{Ai}\;\cdot \;U_{D}\left(S_{A_{i}},S_{D_{2}}\right)=\ldots =\sum_{i=1}^{M}p_{Ai}\;\cdot \;U_{D}\left(S_{A_{i}},S_{D_{N}}\right)\\ \sum_{j=1}^{N}p_{Dj}\;\cdot \;U_{A}\left(S_{A_{1}},S_{D_{j}}\right)=\sum_{j=1}^{N}p_{Dj}\;\cdot \;U_{A}\left(S_{A_{2}},S_{D_{j}}\right)=\ldots =\sum_{j=1}^{N}p_{Dj}\;\cdot \;U_{A}\left(S_{A_{M}},S_{D_{j}}\right) \end{array}$$

If the first approach is taken, changing the strategy of either side of its Nash equilibrium strategy will not increase its profit, so this paper chooses the second approach to solve the Nash equilibrium strategy under the mixed strategy.

Since the probabilities of the adopted strategies are different and the combinations of strategies are varied, the game is consistent with the characteristics of a large scale under a 5G environment. Since the heuristic algorithm has the advantages of fast search and strong merit finding ability [52], intelligent computing is applied to the problem of solving Nash equilibrium under mixed strategy in this game model.

As can be seen from the introduction of Section 2, intelligent computing has the characteristics of high precision and fast speed. It is applied to the model established in this paper. It can solve the mixed strategy Nash equilibrium quickly, accurately, and simply. EO, proposed in 2020, will not quickly converge to an equilibrium state, and it has intermittent balance. Compared with mature algorithms such as PSO, the balance pool used during the period is more easily implemented and more easily jumps out of the local optimum. The calculation is small, and the algorithm effect is good. Therefore, the EO is chosen to solve the problem. To make the result more accurate, applying an adaptive strategy to the algorithm can improve the accuracy and convergence speed, so the AEO is proposed and applied to solve the Nash equilibrium in this section.

The symbols involved in the model and their meanings are listed in Abbreviations.

### 4.3. The Solution of Nash Equilibrium by AEO

In this paper, the AEO is proposed and implemented by improving the EO from three aspects: state partitioning, parameter adaption, and perturbed particle learning.

**Figure 2:**
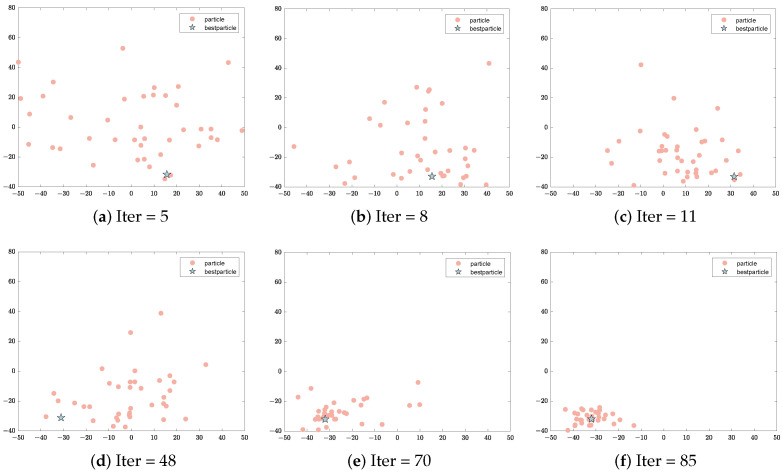
The distribution of the particle population in the EO with the number of iterations.

In the section on state partitioning, the AEO is explored on 23 functions commonly used in CEC for particle distribution characteristics [48]. Some iterative processes are shown in Figure 2. In Figure 2, each plot axis is the horizontal and vertical coordinate points of the two-dimensional interface where the particles are located. And it makes it more intuitive to see the trend of particle positions. The process of particle exploration is shown in Figure 2a. The process of a particle converging toward the best particle is shown in Figure 2b. The process of forming a local convergence is shown in Figure 2c. The process of the best particle jumping out of the current best region is shown in Figure 2d. And the process of exploiting and guiding the particle to converge to the best region again is shown in Figure 2e,f.

For a better description of the state of the whole particle swarm, the states are divided into four types, namely $S_{1}$ (Exploration), $S_{2}$ (Exploitation), $S_{3}$ (Convergence), and $S_{4}$ (Jumping). First, the average distance from each particle to the other particles is calculated by Equation (27).

(27) $$d_{i}=\frac{1}{N_{P}-1}\times \sum_{j=1,j\neq i}^{N_{P}}\sqrt{\sum_{k=1}^{D}{\left(X_{i}^{k}-X_{j}^{k}\right)}^{2}}$$

where $N_{P}$ is the number of particles and *D* is the dimension of the problem. Subsequently, the maximum distance is $d_{max}$, and the minimum distance is $d_{min}$. The best distance $d_{best}$ among them is found and the evolution factor e is calculated from Equation (28).

(28) $$e=\frac{d_{best}-d_{min}}{d_{max}-d_{min}}$$

**Figure 3:**
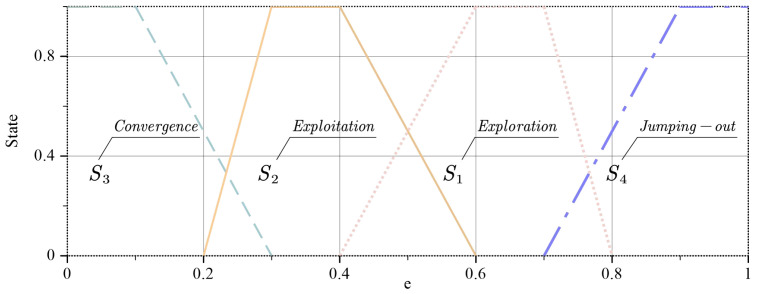
Status segmentation diagram.

Since the motion laws and state distributions of particles in EO and PSO are similar, the fuzzy affiliation degree state distribution in the APSO [48] is used here to classify the population states in AEO. The graph of the particle swarm state distribution with evolution factor is shown in Figure 3.

When *e* is in the affiliation of two states, the state is influenced by the previous state at this time. When *e* is in the interval of $S_{1}$ and $S_{2}$ states, if the previous state is $S_{1}$ or $S_{4}$, then the state is S1 at this time; if the previous state is $S_{2}$ or $S_{3}$, then the state is $S_{2}$ at this time. This change sequence is $S_{1}⟹S_{2}⟹S_{3}⟹S_{4}⟹S_{1}\ldots$.

In the section of parameter adaption, the EO contains three parameters $a_{1}$, $a_{2}$, and GP. $a_{1}$ represents the parameter that controls the exploration capability. $a_{2}$ represents the parameter that manages the exploitation capability. GP plays the role of balancing the exploration and exploitation capabilities.

In this algorithm, *e* is relatively large in the exploitation state and relatively small in the converged state. The variation of GP with e can be calculated by Equation (29). Good robustness of GP in the range of $\left[0.25,0.75\right]$ is proved in the EO. So the variation range of GP in Equation (29) is restricted to $\left[0.3,0.7\right]$.

(29) $$GP=\frac{1}{1+\frac{7}{3}e^{-1.7f}}\in \left[0.3,0.7\right],\;\forall e\in \left[0,1\right]$$

The mechanism of adaptive change with state for the two parameters $a_{1}$ and $a_{2}$ that control the exploration capacity and the exploitation capacity is shown in Table 2.

1. Increasing $a_{1}$ and decreasing $a_{2}$ can help the particles explore their best positions individually without clustering around the local optimal particles.

2. Slightly increasing $a_{1}$ slightly decreasing $a_{2}$. Increasing $a_{1}$ can optimize around the individual optimum, and the optimal solution at this time is likely to be the local optimum rather than the global optimum. Thus, decreasing $a_{2}$ can prevent the particle swarm from falling into the premature convergence problem of the local optimum.

3. A slight increase in $a_{1}$ and an increase in $a_{2}$ allow the particles to converge quickly to the current global optimum position. However, $a_{1}$ should be increased slightly to prevent premature convergence to the wrong local optimum position.

4. Decreasing $a_{1}$ and increasing $a_{2}$ can help particles jump from one optimal position to another global optimal position and lead other particles to move together towards this position.

**Figure 4:**
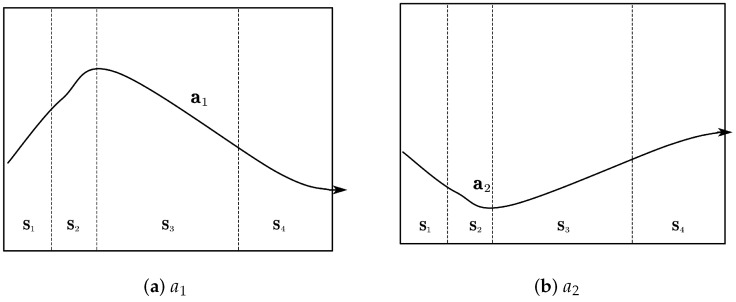
State-based evolution diagram for control parameters $a_{1},a_{2}$.

Figure 4 represents the variation curves of parameters $a_{1},a_{2}$ with state.

In order to prevent the current best particle from being in the local optimal solution, a perturbed particle learning strategy is applied to EO. Interference particle learning is designed to act on the global best particle to help it jump out of the local optimum position at convergence.

Add a Gaussian perturbation to some dimension of the current global optimal particle, as shown in Equation (30). If the particle forms a more optimal solution after the disturbance, other particles can be guided to converge towards it.

(30) $$Ceq_{best}^{d}=Ceq_{best}^{d}+\left(X_{max}^{d}-X_{min}^{d}\right)\;\cdot \;Gaussian\left(\mu ,\sigma^{2}\right)$$

$Ceq_{best}^{d}$ denotes the *i*th dimension. $Gaussian\left(\mu ,\sigma^{2}\right)$ denotes a Gaussian-distributed random number with mean μ of 0 and standard deviation of σ. σ is the elite learning rate, which is calculated as shown in Equation (31).

(31) $$\sigma =\sigma_{max}-\left(\sigma_{max}-\sigma_{min}\right)\;\cdot \;\frac{iter}{MaxIter}$$

The steps of AEO are shown in Algorithm 2.
**Algorithm** **2** Adaptive Equilibrium Optimizer**Require:** ParticleNumber, MaxIter, $C_{max}$, $C_{min}$ **Ensure:** Best Position 1:Initialize the position of the particle swarm using Equation (4)2:Construct the fitness function Fit3:Initialization parameters $a_{1}=2$, $a_{2}=1$, GP=0.54:**for** iter = 1: MaxIter **do**5:     Find the location and concentration of the top 4 best adapted particles $Ceq_{1},Ceq_{2},Ceq_{3},Ceq_{4}$.6:     $C_{eq5}=\left(C_{eq1}+C_{eq2}+C_{eq3}+C_{eq4}\right)/4$7:     $C_{eq,pool}=\left\{C_{eq1},C_{eq2},C_{eq3},C_{eq4},C_{eq5}\right\}$8:     $t={\left(1-\frac{Iter}{MaxIter}\right)}^{a_{2}\frac{Iter}{MaxIter}}$9:     **for** i = 1: ParticleNumber **do**10:          Random selection of a candidate $C_{eq}$ from $C_{eq,pool}$ (state balance pool)11:          Generate random vectors of λ,r12:          Use Equations (6)–(11) to calculate *F*, G·CP, $G_{0}$ and *G*.13:          **if** $Status=S_{4}$ **then**14:              Update $C_{i}=C_{eq1}+\left(C-C_{eq1}\right).F+\frac{G}{\lambda V}\left(1-F\right)$15:          **else**16:              Update $C_{i}=C_{eq}+\left(C-C_{eq}\right).F+\frac{G}{\lambda V}\left(1-F\right)$17:          **end if**18:          Use Equation (27) to calculate the $d_{i}$ of the current particle.19:          Use Equation (28) to calculate the evolution factor *e*.20:          Classify the particle swarm evolutionary state according to Figure 3.21:          The parameters $a_{1},a_{2},GP$ are adjusted according to Table 2 and the evolutionary state of Equation (29).22:          **if** $Status=S_{3}$ **then**23:              $Ceq1^{d}=Ceq1^{d}+\left(X_{max}^{d}-X_{min}^{d}\right)\;\cdot \;Gaussian\left(\mu ,\sigma^{2}\right)$24:            Compare the magnitude of the fitness value of the particle after adding Gaussian perturbation with the current global optimal particle, and update the current global optimal particle position.25:          **end if**26:    **end for**27:    iter = iter+128:**end for**29:Best Position = $C_{eq1}$


## 5. Simulation Experiments and Comparative Analysis

In this section, the offense and defense game simulation experiments are conducted on the established resultant topology diagram of the WSN. The AEO is applied to solve the Nash equilibrium under mixed strategies. The results are analyzed regarding offense and defense strategy selection under different offensive and defensive resources.

### 5.1. Simulation Experiment Model

**Figure 5:**
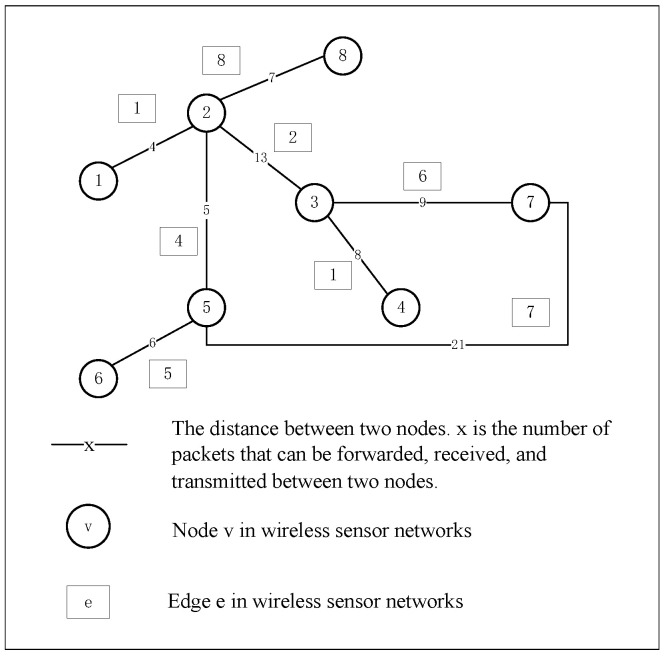
Network topology diagram of the simulation experiment.

The abstract method of the WSN topology diagram shown in Figure 1 in Section 3.1 is applied to the simulation experiment. Combined with the actual structure of the WSN, the simulation experiment diagram with eight WSN nodes and eight communication links is constructed. In this section, a simple WSN topology graph is conducted to study the Nash equilibrium solution problem under mixed strategies in the offense and defense game model. Figure 5 shows the network topology of the WSN.

There are eight nodes and eight edges, the offensive elements of this game model are 16. The importance ranking of nodes and the importance ranking of edges are calculated by Equation (14) and Equation (26), respectively. Table 3 and Table 4 show the importance ranking of nodes and edges, respectively. By calculating the importance of nodes and edges, the results are better analyzed. The change of offense and defense probability to important nodes and links can be analyzed, so as to better analyze the changes in offensive and defensive behavior.

When the offensive and defensive resources are assumed to be 3, 6, 7, 11, and 14, the strategies for the offender are $C_{16}^{3}=560$, $C_{16}^{7}=11,440$, $C_{16}^{6}=8008$, $C_{16}^{11}=4368$, and $C_{16}^{14}=120$, respectively. Therefore, the scale of the offense and defense games in this simulated network topology diagram can meet the large-scale characteristics of the 5G environment.

### 5.2. Simulation Experiment Tools and Parameters

The simulation experiment environment is shown in Table 5. According to the network topology that Figure 5 established, the offensive and defensive resources are changed to conduct simulation experiments. The offensive resources represent the number of nodes and edges that can be attacked. The defensive resources represent the number of nodes and edges that can be attacked. The specific experimental parameters and scale are shown in Table 6. (Note: NOR represents the number of offensive resources, and NOS represents the number of offensive strategies. NDR represents the number of defensive resources, and NDS represents the number of defensive strategies. SOD represents the scale of offense and defense.)

As can be seen from Table 6, the control variable method is adopted to control a single variable, such as keeping the offensive resources unchanged and changing the defensive resources, so as to carry out the simulation confrontation of the offensive and defensive game. By solving the Nash equilibrium, the change of the defense and offense behavior of both the offensive and defensive parties to the nodes and edges in the established model is analyzed. And Table 6 shows that the scale of the offensive and defensive game has reached millions or even tens of millions of levels, which can meet the large-scale characteristics of the 5G environment.

### 5.3. Solving Nash Equilibrium

The performance of AEO is compared with EO, BA, DE, PSO, and WOA among 23 functions commonly used in CEC; these 23 functions are described in the literature [41]. And the comparison results are shown in Table 7. > indicates that the current algorithm outperforms the AEO with this function. < indicates that the AEO outperforms the current algorithm with this function. = indicates that the AEO and the current algorithm have the same performance under this function. The last row of the table counts the number of functions whose performance of AEO is equal or superior to that of other algorithms.

Among the 23 sets of commonly measured functions of CEC, $f_{1}-f_{7}$ are single-peaked functions, $f_{8}-f_{13}$ are multi-peaked functions, and $f_{14}-f_{23}$ are mixed functions. As can be seen from Table 7, the AEO has improved algorithm performance compared with other algorithms, especially compared with the PSO, BA, and WOA.

For functions $f_{1}-f_{5},f_{7},f_{9}-f_{11},f_{13}-f_{14},f_{16}-f_{19},f_{21}$, the AEO outperforms or equals the other algorithms. For the remaining functions, the AEO may under-perform compared to one or several algorithms, but the difference is not significant. It can be seen from Table 7 that among the 23 test functions, AEO performs well in more than 80% of the functions compared with the original algorithm and other algorithms.

**Figure 6:**
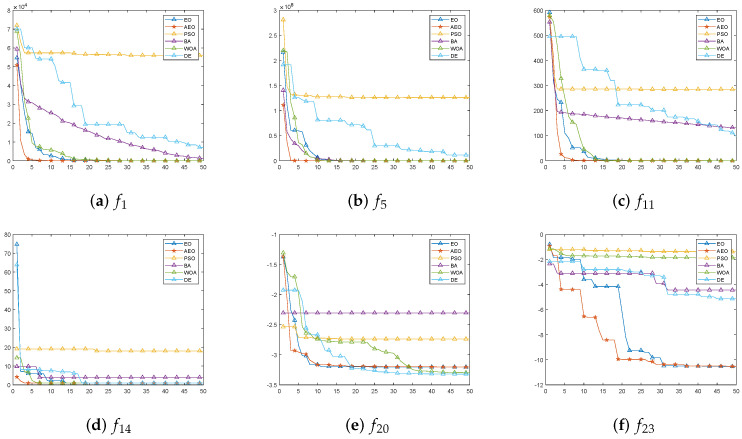
Comparison of the convergence speed of different algorithms under different functions.

The convergence speed of these algorithms is compared. The first 50 iterations of particles in some functions are selected for image visualization, as shown in Figure 6. It can be seen from the Figure 6 that the convergence speed of AEO has obvious advantages in the early stage of iteration. It can find the optimal solution more quickly. This is because the Gaussian disturbances and parameter optimization strategy are applied to AEO. This is because the three adaptive strategies help particles quickly jump out of local optimal solutions to find the optimal solution in the global scope. The perturbed particle learning can further optimize the global optimal solution found by the parameter adaptation strategy. Thus, the accuracy of AEO is improved.

Dimension $D_{1}$ is the number of strategies that offender *A* can take and $D_{2}$ is the number of strategies that defender *D* can take. Therefore $D_{1}=M,D_{2}=N$. In this model, Equation (26) can be used as $fitness_{A}$ and $fitness_{D}$ function set by applying the AEO solution.

It can be seen from Equation (26) that the smaller the values of $fitness_{A}$ and $fitness_{D}$, the more stable the returns. The Nash equilibrium strategy solved by AEO is applied to the offensive and defensive games, the network topology graph is attacked 10 times randomly, and the gain of the defender is calculated. Under the same offensive and defensive resources, the gain of the defender changes with the times of offenses, as shown in Table 8.

The model is a zero-sum game model, and the gain of offenders is consistent with the gain of defenders. Table 8 shows that the gain of the defender is stable under 10 random offenses, and its mean square deviation is 0.005. Therefore, it is feasible to use AEO to solve the Nash equilibrium under mixed strategies.

### 5.4. Offensive Strategy Selection under Different Defense Strategies

**Figure 7:**
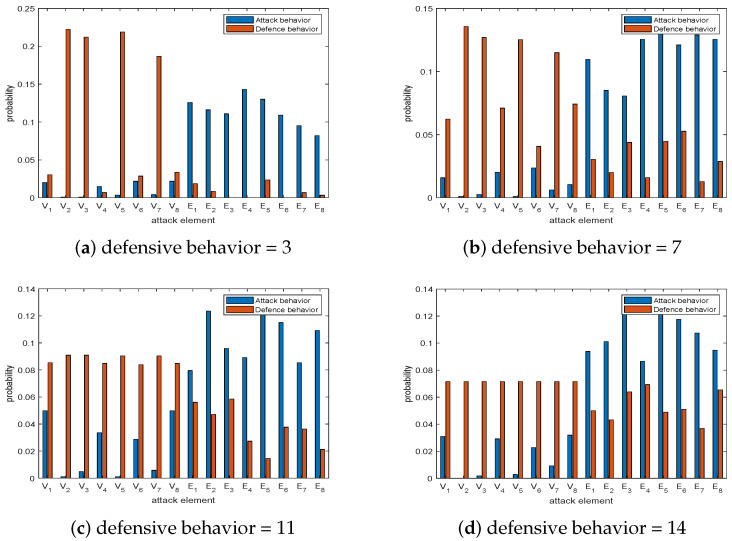
Offense and defense game with 6 offensive resources and different defensive resources.

The offensive resources are set to 6 and the defensive resources are set to 3, 7, 11, and 14 for the offense and defense game. After solving the Nash equilibrium in this game model using the AEO, the results are visualized and mapped to the probabilities of attacking and defending elements, as shown in Figure 7.

As can be seen from Figure 7a, when the defensive resources are small, the offender will attack the communication link with a high probability. The nodes $E_{4},E_{5},E_{6}$, and $E_{1}$ with a high probability are attacked, and they are low in terms of link importance. The defender will defend the node part with a high probability, especially the node of higher importance, such as $V_{2},V_{3},V_{5}$. When the defense resources increase slightly, the defense direction of the defender changes to defend the important nodes and edges. The offender starts to attack the important edges and the unimportant nodes, and the probability of attacking the nodes can be seen in Figure 7c,d. When the defensive resources continue to increase, the defender will defend each element with equal probability. For the offender, the probability of attacking the communication link will gradually increase. The probability of attacking an important link, such as $E_{5},E_{7}$, will be increased to achieve the Nash equilibrium of returns.

### 5.5. Defensive Strategy Selection under Different Offense Strategies

**Figure 8:**
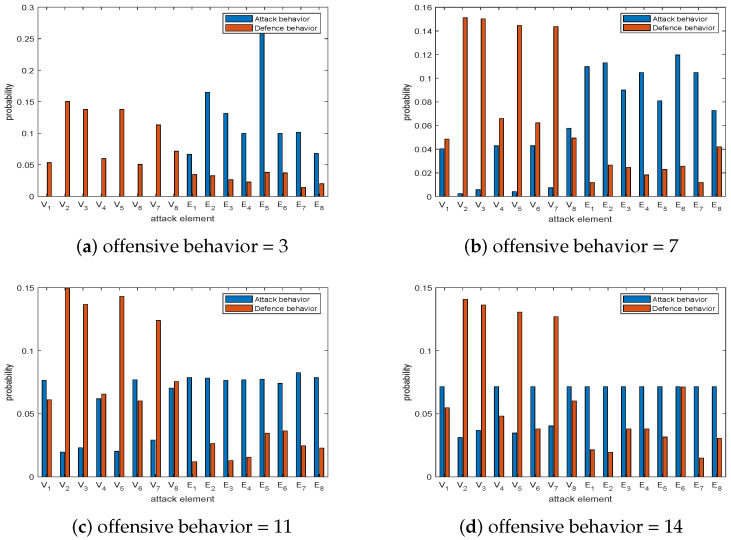
Offense and defense game with 6 defensive resources and different offensive resources.

The defensive resources are set as 6, and the offensive resources of the offense and defense game are set to 3, 7, 11, and 14. After solving the Nash equilibrium in this game model with the AEO, the results are visualized and is mapped to the probabilities of attacking and defending elements, as shown in Figure 8.

The defensive resources are fixed and the offensive resources are relatively small; the results can be seen in Figure 8a. The offender mainly attacks the important edges. The defender mainly defends the nodes in the network and defends the important nodes with maximum probability. The results when the offensive resources increase slightly can be seen in Figure 8b. The offense direction of offenders changes to attack the edges and the unimportant nodes, such as $V_{1},V_{4},V_{6},V_{8}$. But the probability of attacking the edges is greater than the probability of attacking the nodes when the defense is still focused on the important node part. The results of Figure 8c,d show that when the attack resources continue to increase, the defender defends important nodes, such as $V_{2},V_{3},V_{5}$, and communication links to win the game. The attack probability of the offender on the unimportant node $V_{1}$ and $V_{4},V_{6}$ increases and tends to be consistent. Finally, the offender will attack the communication link and the unimportant node with the same probability.

## 6. Conclusions

A complete information static game model is proposed to solve the offense and defense confrontation problem in 5G WSNs. Due to the large number of combat strategies under the mixed strategy combination, the AEO is proposed to solves the Nash equilibrium in the game. The AEO and other heuristic algorithms are tested in CEC, in 23 test functions, to compare their performances. The experimental results show that AEO has better optimization ability and high precision. Under different offensive and defensive resources, the behavior analysis and strategy selection of the two players in the game are simulated. The experimental results show that the offender will attack the important elements when the offensive resources are small. When the offensive resources are greater, the offender will attack the link indiscriminately. When the defense resources are small, the defender will defend the elements which are of high importance with high probability. When there are more defense resources, the defender should allocate the same resources to defend every element. The research in this paper can provide a certain theoretical research and analysis method for resource allocation and defense behavior in the offense and defense game of 5G WSNs.

## Figures and Tables

**Table 1:** Matrix of gains for both offenders and defenders.

	$S_{D1}$	$S_{D2}$	…	$S_{DN}$
$S_{A1}$	$U_{A_{11}},U_{D_{11}}$	$U_{A_{12}},U_{D_{12}}$	…	$U_{A_{1N}},U_{D_{1N}}$
$S_{A2}$	$U_{A_{21}},U_{D_{21}}$	$U_{A_{22}},U_{D_{22}}$	…	$U_{A_{2N}},U_{D_{2N}}$
⋮	⋮	⋮	⋮	⋮
$S_{AM}$	$U_{A_{M1}},U_{D_{M1}}$	$U_{A_{M2}},U_{D_{M2}}$	…	$U_{A_{MN}},U_{D_{MN}}$

**Table 2:** Table of adaptive changes of $a_{1}$ and $a_{2}$ with state.

Status	$a_{1}$	$a_{2}$
$S_{1}$	Increase	Decrease
$S_{2}$	Increase Slightly	Decrease
$S_{3}$	Increase Slightly	Increase
$S_{4}$	Decrease	Increase

**Table 3:** The importance of nodes.

*N*	$V_{1}$	$V_{2}$	$V_{3}$	$V_{4}$	$V_{5}$	$V_{6}$	$V_{7}$	$V_{8}$
*I*	4	29	30	8	32	6	30	7
sort	$V_{5}>V_{3}=V_{7}>V_{2}>V_{4}>V_{6}>V_{8}>V_{1}$							

**Table 4:** The importance of edges.

*N*	$E_{1}$	$E_{2}$	$E_{3}$	$E_{4}$	$E_{5}$	$E_{6}$	$E_{7}$	$E_{8}$
*I*	0.277	0.289	0.332	0.179	0.304	0.234	0.398	0.318
sort	$E_{7}>E_{3}>E_{8}>E_{5}>E_{2}>E_{1}>E_{6}>E_{4}$							

**Table 5:** Simulation experiment environment.

Operating System	Windows 11
Processor	Intel(R) Core(TM) i5-8500 CPU @ 3.00 GHz
RAM	16 GB
Simulation tool	Matlab
Matlab version	9.11.0.1769968 (R2021b)

**Table 6:** The parameter of experiment.

NOR	NOS	NDR	NDS	SOD
6	$C_{16}^{6}=8008$	3	$C_{16}^{3}=560$	4,484,480
6	$C_{16}^{6}=8008$	7	$C_{16}^{7}=11,440$	91,611,520
6	$C_{16}^{6}=8008$	11	$C_{16}^{11}=4368$	34,978,944
6	$C_{16}^{6}=8008$	14	$C_{16}^{14}=120$	960,960
3	$C_{16}^{3}=560$	6	$C_{16}^{6}=8008$	4,484,480
7	$C_{16}^{7}=11,440$	6	$C_{16}^{6}=8008$	91,611,520
11	$C_{16}^{11}=4368$	6	$C_{16}^{6}=8008$	34,978,944
14	$C_{16}^{14}=120$	6	$C_{16}^{6}=8008$	960,960

**Table 7:** AEO and other intelligent algorithms’ performance comparison.

F	AEO	BA	DE	EO	PSO	WOA
$f_{1}$	$7.11\times 10^{-129}\left(=\right)$	$6.48\times 10^{0}\left(<\right)$	$1.45\times 10^{-4}\left(<\right)$	$1.17\times 10^{-48}\left(<\right)$	$4.00\times 10^{4}\left(<\right)$	$5.20\times 10^{-86}\left(<\right)$
$f_{2}$	$2.29\times 10^{-66}\left(=\right)$	$3.94\times 10^{33}\left(<\right)$	$4.64\times 10^{-2}\left(<\right)$	$9.38\times 10^{-27}\left(<\right)$	$3.94\times 10^{37}\left(<\right)$	$9.46\times 10^{-53}\left(<\right)$
$f_{3}$	$1.14\times 10^{-106}\left(=\right)$	$5.35\times 10^{1}\left(<\right)$	$2.65\times 10^{4}\left(<\right)$	$3.72\times 10^{-11}\left(<\right)$	$7.46\times 10^{4}\left(<\right)$	$2.99\times 10^{4}\left(<\right)$
$f_{4}$	$2.36\times 10^{-60}\left(=\right)$	$1.07\times 10^{1}\left(<\right)$	$1.03\times 10^{1}\left(<\right)$	$1.79\times 10^{-12}\left(<\right)$	$7.04\times 10^{1}\left(<\right)$	$2.78\times 10^{1}\left(<\right)$
$f_{5}$	$2.42\times 10^{1}\left(=\right)$	$1.86\times 10^{3}\left(<\right)$	$8.72\times 10^{1}\left(<\right)$	$2.48\times 10^{1}\left(<\right)$	$1.11\times 10^{8}\left(<\right)$	$2.75\times 10^{1}\left(<\right)$
$f_{6}$	$1.18\times 10^{-5}\left(=\right)$	$6.34\times 10^{0}\left(<\right)$	$1.35\times 10^{-4}\left(<\right)$	$1.46\times 10^{-7}\left(>\right)$	$4.47\times 10^{4}\left(<\right)$	$7.98\times 10^{-2}\left(<\right)$
$f_{7}$	$1.36\times 10^{-4}\left(=\right)$	$4.28\times 10^{1}\left(<\right)$	$4.97\times 10^{-2}\left(<\right)$	$8.15\times 10^{-4}\left(<\right)$	$5.00\times 10^{1}\left(<\right)$	$2.17\times 10^{-3}\left(<\right)$
$f_{8}$	$-8.81\times 10^{3}\left(=\right)$	$-5.91\times 10^{89}\left(>\right)$	$-1.13\times 10^{4}\left(>\right)$	$-9.22\times 10^{3}\left(>\right)$	$-3.78\times 10^{3}\left(<\right)$	$-1.13\times 10^{4}\left(>\right)$
$f_{9}$	$0.00\times 10^{0}\left(=\right)$	$2.75\times 10^{2}\left(<\right)$	$9.21\times 10^{1}\left(<\right)$	$0.00\times 10^{0}\left(=\right)$	$3.41\times 10^{2}\left(<\right)$	$5.68\times 10^{-15}\left(<\right)$
$f_{10}$	$1.48\times 10^{-15}\left(=\right)$	$1.02\times 10^{1}\left(<\right)$	$3.43\times 10^{-3}\left(<\right)$	$7.88\times 10^{-15}\left(<\right)$	$1.94\times 10^{1}\left(<\right)$	$4.20\times 10^{-15}\left(<\right)$
$f_{11}$	$0.00\times 10^{0}\left(=\right)$	$3.77\times 10^{-1}\left(<\right)$	$4.53\times 10^{-3}\left(<\right)$	$0.00\times 10^{0}\left(=\right)$	$4.05\times 10^{2}\left(<\right)$	$2.75\times 10^{-3}\left(<\right)$
$f_{12}$	$5.62\times 10^{-7}\left(=\right)$	$2.15\times 10^{1}\left(<\right)$	$2.48\times 10^{-5}\left(<\right)$	$4.45\times 10^{-9}\left(>\right)$	$2.01\times 10^{8}\left(<\right)$	$1.21\times 10^{-2}\left(<\right)$
$f_{13}$	$1.27\times 10^{-5}\left(=\right)$	$1.03\times 10^{0}\left(<\right)$	$8.46\times 10^{-5}\left(<\right)$	$1.52\times 10^{-2}\left(<\right)$	$5.44\times 10^{8}\left(<\right)$	$2.12\times 10^{-1}\left(<\right)$
$f_{14}$	$9.98\times 10^{-1}\left(=\right)$	$2.90\times 10^{0}\left(<\right)$	$1.06\times 10^{0}\left(<\right)$	$9.98\times 10^{-1}\left(=\right)$	$1.02\times 10^{1}\left(<\right)$	$2.50\times 10^{0}\left(<\right)$
$f_{15}$	$2.37\times 10^{-3}\left(=\right)$	$1.17\times 10^{-3}\left(>\right)$	$2.72\times 10^{-3}\left(<\right)$	$1.74\times 10^{-3}\left(>\right)$	$3.73\times 10^{-2}\left(<\right)$	$6.00\times 10^{-4}\left(>\right)$
$f_{16}$	$-1.03\times 10^{0}\left(=\right)$	$-1.03\times 10^{0}\left(<\right)$	$-1.03\times 10^{0}\left(=\right)$	$-1.03\times 10^{0}\left(=\right)$	$-8.69\times 10^{-1}\left(<\right)$	$-1.03\times 10^{0}\left(<\right)$
$f_{17}$	$3.98\times 10^{-1}\left(=\right)$	$3.98\times 10^{-1}\left(<\right)$	$3.98\times 10^{-1}\left(=\right)$	$3.98\times 10^{-1}\left(=\right)$	$6.69\times 10^{-1}\left(<\right)$	$3.98\times 10^{-1}\left(<\right)$
$f_{18}$	$3.00\times 10^{0}\left(=\right)$	$3.05\times 10^{0}\left(<\right)$	$3.00\times 10^{0}\left(=\right)$	$3.00\times 10^{0}\left(=\right)$	$1.04\times 10^{1}\left(<\right)$	$3.00\times 10^{0}\left(<\right)$
$f_{19}$	$-3.86\times 10^{0}\left(=\right)$	$-3.82\times 10^{0}\left(<\right)$	$-3.86\times 10^{0}\left(=\right)$	$-3.86\times 10^{0}\left(=\right)$	$-3.76\times 10^{0}\left(<\right)$	$-3.86\times 10^{0}\left(<\right)$
$f_{20}$	$-3.25\times 10^{0}\left(=\right)$	$-2.55\times 10^{0}\left(<\right)$	$-3.27\times 10^{0}\left(>\right)$	$-3.26\times 10^{0}\left(>\right)$	$-2.19\times 10^{0}\left(<\right)$	$-3.21\times 10^{0}\left(<\right)$
$f_{21}$	$-9.14\times 10^{0}\left(=\right)$	$-5.15\times 10^{0}\left(<\right)$	$-8.14\times 10^{0}\left(<\right)$	$-8.80\times 10^{0}\left(<\right)$	$-1.34\times 10^{0}\left(<\right)$	$-8.79\times 10^{0}\left(<\right)$
$f_{22}$	$-1.04\times 10^{1}\left(=\right)$	$-5.57\times 10^{0}\left(<\right)$	$-1.05\times 10^{1}\left(>\right)$	$-1.05\times 10^{1}\left(>\right)$	$-1.56\times 10^{0}\left(<\right)$	$-8.65\times 10^{0}\left(<\right)$
$f_{23}$	$-9.82\times 10^{0}\left(=\right)$	$-4.82\times 10^{0}\left(<\right)$	$-1.02\times 10^{1}\left(>\right)$	$-9.32\times 10^{0}\left(<\right)$	$-1.93\times 10^{0}\left(<\right)$	$-7.50\times 10^{0}\left(<\right)$
ine Comparison of results:		≤:21	≤:19	≤:18	≤:23	≤:21
ine						

**Table 8:** Gain of the defender changes with the times of offenses.

time	1	2	3	4	5
gain of the defender	−2.702	−2.702	−2.705	−2.698	−2.708
time	6	7	8	9	10
gain of the defender	−2.714	−2.698	−2.703	−2.702	−2.700

## Data Availability

The data used to support the findings of this study are included within the article.

## Abbreviations

The following abbreviations are used in this manuscript:

*G*	the topology diagram of WSN
$G_{max}$	Maximum connectivity of topology diagram G
*V*	the set of nodes
*E*	the set of edges
*A*	the diagonal matrix of graph G
$a_{ij}$	information transfer link situation of nodes $V_{i}$ to $V_{j}$
$S_{ij}$	the number of packets transmitted, received and forwarded between node $V_{i}$ to $V_{j}$
$I_{V_{i}}$	the important of the node $V_{i}$
$I_{E_{i}}$	the important of the edge $E_{i}$
*Q*	the total number of offensive and defensive resources
$Q_{A}$	number of resources that the offender can offense
$Q_{D}$	number of resources that the defender can defend
$S_{A}$	the set of strategies for offense
$S_{D}$	the set of strategies for defense
*M*	the number of offensive strategies
*N*	the number of defensive strategies
$A_{V_{i}}$	the success of attack on node $V_{i}$
$U_{A}$	the set of revenue of the offender
$U_{D}$	the set of revenue of the defender
$S_{A}^{*}$	the optimal offense strategies under the offense and defense game
$S_{D}^{*}$	the optimal defense strategies under the offense and defense game
$P_{A}$	the probability of the offender taking each strategy
$P_{D}$	the probability of the defender taking each strategy
$U_{A}^{{}^{\prime }}$	the revenue of the offender under mixed strategy
$U_{D}^{{}^{\prime }}$	the revenue of the defender under mixed strategy
$G^{{}^{\prime }}$	the topology diagram of WSN after offense-defense game
$G_{max}^{{}^{\prime }}$	Maximum connectivity of topology diagram $G^{{}^{\prime }}$
